# Supplementary material for: Insufficient social distancing may contribute to COVID-19 outbreak: The case of Ijuí city in Brazil
Source: PLoS One. 2021 Feb 17;16(2):e0246520. doi: 10.1371/journal.pone.0246520 (PMC7888680; doi:10.1371/journal.pone.0246520)
Supplement: S1 File — (PDF) [file pone.0246520.s001.pdf]

Insufficient social distancing may contribute to COVID-19 outbreak: the case of Ijuí city in Brazil

Thiago Gomes Heck[1,2,3,4*¶], Rafael Z. Frantz[4,5¶], Matias Nunes Frizzo[1,2,3], Carlos Henrique Ramires François[3], Mirna Stela Ludwig[1,2,3], Marilia Arndt Mesenburg[6,7], Giovano Pereira Buratti[2],

Lígia Beatriz Bento Franz[2,8], Evelise Moraes Berlezi[2,3,8]

[1] Research Group in Physiology, Regional University of Northwestern Rio Grande do Sul State, Ijuí, Rio Grande do Sul State, Brazil.
[2] Postgraduate Program in Integral Attention to Health, Regional University of Northwestern Rio Grande do Sul State, Ijuí, Rio Grande do Sul State, Brazil.
[3] Medicine Course, Department of Life Sciences, Regional University of Northwestern Rio Grande do Sul State, Ijuí, Rio Grande do Sul State, Brazil.
[4] Postgraduate Program in Mathematical and Computational Modeling, Ijuí, RS, Brazil.
[5] Department of Exact Sciences and Engineering, Regional University of Northwestern Rio Grande do Sul State, Ijuí, Rio Grande do Sul State, Brazil. [6] Federal University of Pelotas (UFPel), Pelotas, Rio Grande do Sul State, Brazil.
[7] Federal University of Health Sciences of Porto Alegre (UFCSPA), Porto Alegre, Rio Grande do Sul State, Brazil.
[8] Research Group in Human Aging, Regional University of Northwestern Rio Grande do Sul State, Ijuí, Rio Grande do Sul State, Brazil.

***Corresponding author**

Thiago Gomes Heck, thiago.heck@unijui.edu.br

Regional University of Northwestern Rio Grande do Sul State (UNIJUI)

Rua do Comércio, 3000, Bairro Universitário, Ijuí, RS 98700-000, Brazil

Supplementary Figure S1. Distribution of COVID-19 cases in Urban area of Ijuí in June, August and November.

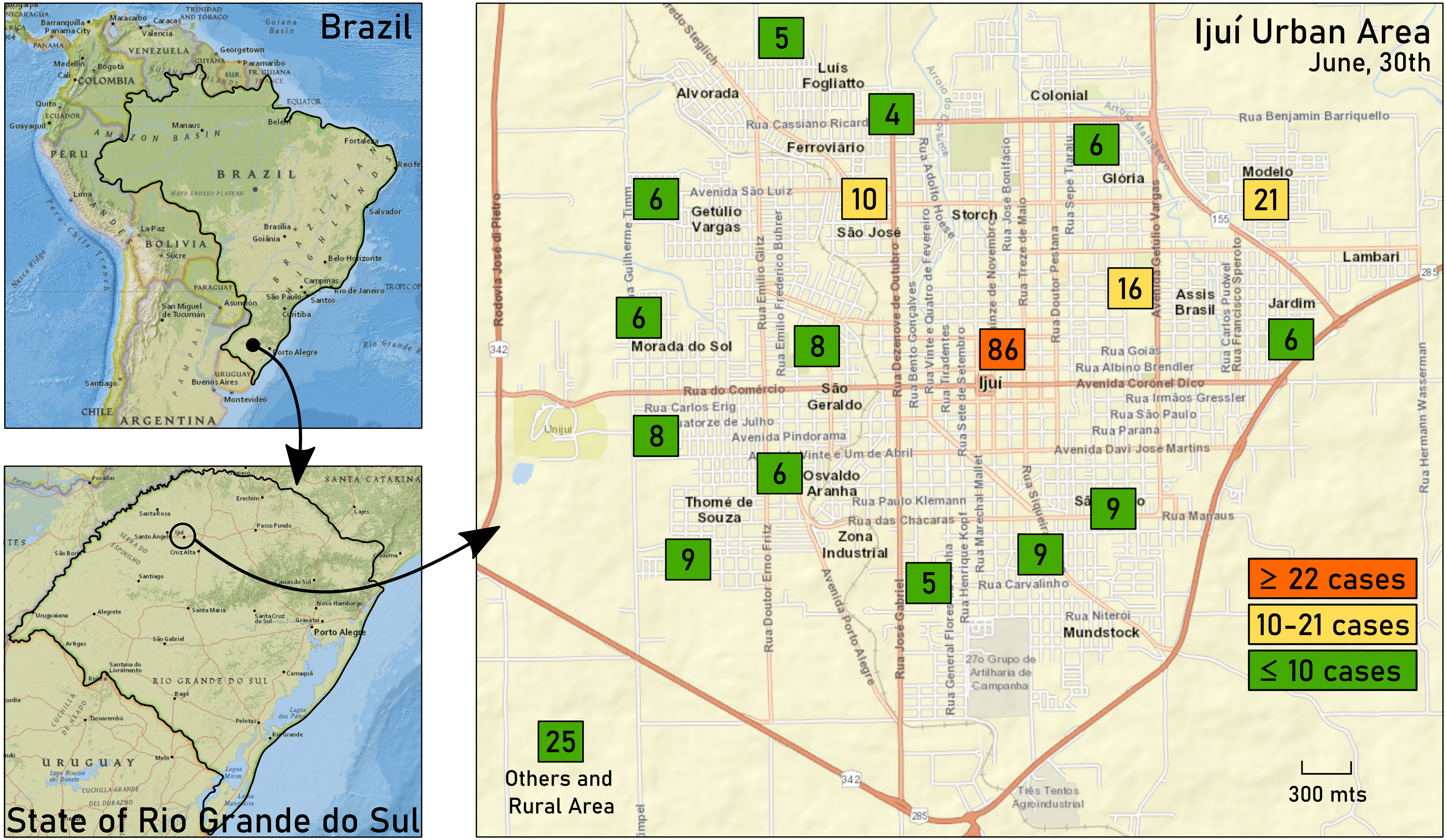

Maps source: U.S. Geological Survey web site
https://viewer.nationalmap.gov/advanced-viewer

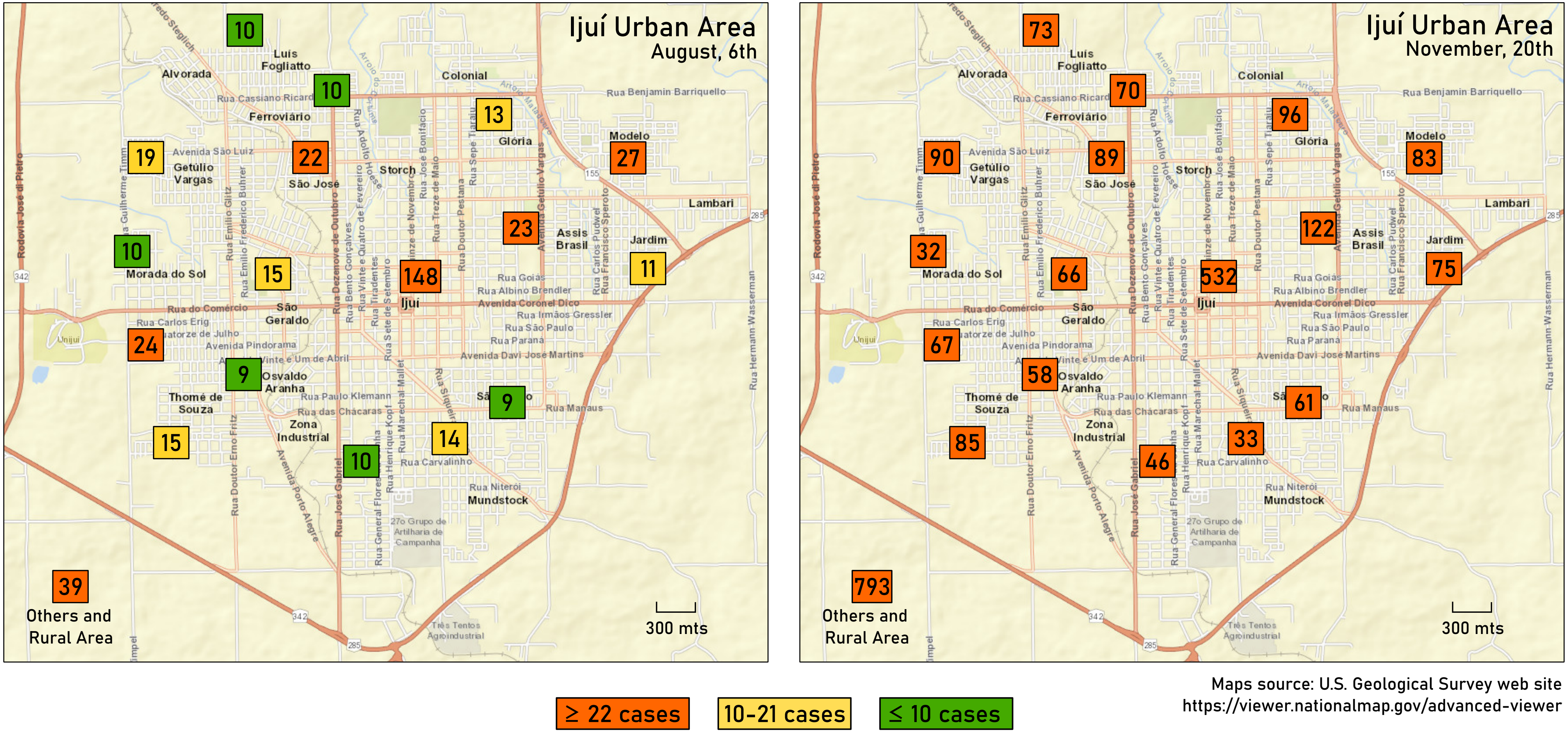

Maps source: U.S. Geological Survey web site
https://viewer.nationalmap.gov/advanced-viewer

QUESTIONNAIRE

1- FU: (IBGE FU code)

____

2- ROUND:

____

3- CITY CODE:

____

4- Sector: (TWO DIGITS)

____

5- Sequential number of the interview in the sector: (TWO DIGITS)

____

6- Interviewee's household number: (TWO DIGITS) (ACCORDING TO ADDRESS LIST)

____

7- Number of regular residents in the household ○ 1 resident ○ 2 residents ○ 3 residents ○ 4 residents ○ 5 residents ○ 6 residents ○ 7 residents ○ 8 residents ○ 9 residents ○ 10 residents ○ 11 residents ○ 12 residents ○ 13 residents ○ 14 residents ○ 15 residents

8- Number of the selected resident: (According to resident board) __________________________

9- Name of the selected resident:

____

10- Number of the resident who answers the questionnaire (Consult the resident board)

____

11- Who answers the questionnaire? ○ The person him/herself ○ Mother ○ Father ○ Guardian or caregiver

12- Gender of (NAME):
- ○ Male ○ Female

13- What is <NAME>'s date of birth?
__ __ / __ __ / __ __ __ __ (day/month/year)

14- How old is (NAME)? (If less than 1 year, put 0)
____

15- Did (NAME) attend school?
- ○ Yes
- ○ No

16- (NAME'S) education level: ○ Preschool education ○ Incomplete primary school ○ Complete primary school ○ Incomplete secondary school ○ Complete secondary school ○ Incomplete college/university ○ Complete college/university
- ○ Graduate school

17- What was the last grade that (NAME) completed in school?
____

18- What is the education level of the resident who studied the most? ○ Illiterate/did not study ○ Preschool education ○ Incomplete primary school ○ Complete primary school ○ Incomplete secondary school ○ Complete secondary school ○ Incomplete college/university ○ Complete college/university
- ○ Graduate school

19- What was the last grade completed by the resident who studied the most in this household?
____

20- How does (NAME) self-identify in terms of color or race? ○ White ○ Brown ○ Black ○ Yellow ○ Indigenous
OBS: this is the standard question used in the Brazilian census

21- In the last two weeks, that is, since (DAY OF THE WEEK) of the week before last, did (NAME) have FEVER: ○ Yes ○ No

22- How many days ago did it start?

____

23- Do you still have a fever? ○ Yes

○ No

24- For how many days did the fever last?

____

25- In the last two weeks, that is, since (DAY OF THE WEEK) of the week before last, did (NAME) have a SORE THROAT: ○ Yes ○ No

26- How many days ago did it start?

____

27- Do you still have a sore throat? ○ Yes

○ No

28- For how many days did the sore throat last?

____

29- In the last two weeks, that is, since (DAY OF THE WEEK) of the week before last, did (NAME) have a COUGH: ○ Yes ○ No

○ Always has it/ constantly

30- Was it a wet cough? ○ Yes ○ No

31- How many days ago did it start?

____

32- Do you still have a cough? ○ Yes

○ No

33- For how many days did the cough last?

____

34- In the last two weeks, that is, since (DAY OF THE WEEK) of the week before last, did (NAME) have DIFFICULTY BREATHING: ○ Yes ○ No

- Always has it/constantly

35- Was your nose blocked?

- Yes ○ No

36- How many days ago did it start?

____

37- Do you still have difficulty breathing? ○ Yes

- No

38- For how many days did the difficulty breathing last?

____

39- In the last two weeks, that is, since (DAY OF THE WEEK) of the week before last, did (NAME) have HEART PALPITATIONS, A RACING OR IRREGULAR HEARTBEAT:

- Yes ○ No
- Always has them/ constantly

40- How many days ago did they start?

____

41- Do you still have this heart problem? ○ Yes

- No

42- For how many days did the heart palpitations last?

____

43- In the last two weeks, that is, since (DAY OF THE WEEK) of the week before last, did (NAME) start to have TROUBLE SMELLING OR TASTING THINGS? ○ Yes

- No

44- How many days ago did it start?

____

45- Do you still have this problem? ○ Yes ○ No

46- For how many days were you unable to smell or taste things?

____

47- In the last two weeks, that is, since (DAY OF THE WEEK) of the week before last, did (NAME) have DIARRHEA: ○ Yes

- ○ No

48- How many days ago did it start?

____

49- Do you still have diarrhea?

- ○ Yes
- ○ No

50- For how many days did the diarrhea last?

____

51- In the last two weeks, that is, since (DAY OF THE WEEK) of the week before last, did (NAME) suffer VOMITING: ○ Yes

- ○ No

52- How many days ago did it start?

____

53- Do you still suffer vomiting? ○ Yes ○ No

54- For how many days did the vomiting last?

____

55- Has any doctor ever said that (NAME) has:

	Yes	No
Hypertension or high blood pressure	○	○
Diabetes or high blood sugar	○	○
Asthma or bronchitis	○	○
Cancer (any type)	○	○

Chronic kidney disease	○	○
Any heart disease	○	○
Another chronic disease	○	○

56- Did (NAME) seek a health service in the last 15 days?
- ○ Yes
- ○ No

57- What kind of health service?
- ○ Health center ○ Emergency room ○ Private doctor ○ Hospital
- ○ Other health service

58- Why did you seek the service? ○ Vaccination ○ Preexisting health problem ○ Scheduled return visit ○ Flu symptoms or sore throat ○ To obtain medication
- ○ Other reason

59- Regarding the social distancing that is being recommended by health authorities, that is, staying at home and avoiding contact with other people, how much does (NAME) think he/she is managing to do? (read alternatives)
- ○ Very little
- ○ Little
- ○ More or less
- ○ A lot
- ○ Practically isolated from everyone

60- What has (NAME)'s activity routine been like? (read alternatives) ○ Stay home all the time
- ○ Go out only for essential things like buying food ○ Go out from time to time to shop and stretch your legs ○ Go out every day for some activity
- ○ Go out every day, all day, for work or other regular activity

61- Thinking about the home routine, who enters the house? (read alternatives)
- ○ Only family members who live together, if any, and no one else ○ Some close relatives visit 1-2 times a week ○ Some close relatives visit almost every day ○ Friends, relatives, or others who visit 1-2 times a week ○ Friends, relatives, or others who visit almost every day

62- TEST IDENTIFICATION: (NUMBER: ROUND + MUNICIPALITY + SECTOR + HOUSEHOLD) (READ OUT LOUD)

____

63- TEST RESULT: ○ POSITIVE ○ NEGATIVE
○ INCONCLUSIVE

64- IF POSITIVE, MARK THE RESIDENTS TO BE TESTED: (SEE HOUSEHOLD TABLE) ○ Resident 1 ○ Resident 2 ○ Resident 3 ○ Resident 4 ○ Resident 5 ○ Resident 6 ○ Resident 7 ○ Resident 8 ○ Resident 9 ○ Resident 10 ○ Resident 11 ○ Resident 12 ○ Resident 13 ○ Resident 14 ○ Resident 15

65- Interviewee name:
____

66- Contact phone:
( ) _______-_______

QUESTIONÁRIO

1 - UF: (código da UF IBGE)

____

2 - Rodada:

____

3 - Cidade: (Código)

____

4 - Setor: (DOIS DÍGITOS)

____

5 - Número sequencial da entrevista no setor: (DOIS DÍGITOS)

____

6 - Número do domicílio do entrevistado: (DOIS DÍGITOS)

____

7 - Número de moradores regulares no domicílio:

- 1 morador
- 2 moradores
- 3 moradores
- 4 moradores
- 5 moradores
- 6 moradores
- 7 moradores
- 8 moradores
- 9 moradores
- 10 moradores
- 11 moradores
- 12 moradores
- 13 moradores
- 14 moradores
- 15 moradores

8 - Número do morador selecionado: (Consultar quadro de moradores)

____

9 - Nome do morador selecionado:

____

10 - Nº do morador entrevistado (Consultar quadro de moradores):

____

11 - Quem responde o questionário?

- A própria pessoa
- Mãe
- Pai
- Outro responsável ou cuidador

12 - Sexo de (NOME):

- Masculino
- Feminino

13 - Qual a data de nascimento de (NOME)? (dd/mm/aaaa)

_/_/______

14 - Qual idade de (NOME)? (Se for menor que 1 ano, colocar 0)

____

15 - (NOME) frequentou escola?

- Sim
- Não

16 - Grau de escolaridade de (NOME):

- Educação infantil
- Fundamental incompleto
- Fundamental completo
- Médio incompleto

Médio completo Superior incompleto Superior completo Pós graduação **17 - Qual foi a última série/ano que (NOME) completou na escola?**

____

18 - Qual o grau escolaridade do morador que mais estudou?

Analfabeto/não estudou Educação infantil

Fundamental incompleto Fundamental completo Médio incompleto

Médio completo Superior incompleto Superior completo Pós graduação **19 - Qual foi a última série/ano completada pelo morador que mais estudou nesta casa?**

____

20 - Como (NOME) se classifica em termos de cor ou raça?

Branca Parda

Preta Amarela Indígena

21- Nas últimas duas semanas, isto é, desde (DIA DA SEMANA) da semana retrasada, (NOME) teve FEBRE:

Sim Não

21 - Começou há quantos dias?

____

22 - Ainda tem febre?

Sim Não **23 - Quantos dias durou a febre?**

24 - Nas últimas duas semanas, isto é, desde (DIA DA SEMANA) da semana retrasada, (NOME) DOR DE GARGANTA:

◉ Sim ◉ Não

◉ Tem sempre/constantemente

25 - Começou há quantos dias?

26 - Ainda tem dor de garganta?

◉ Sim ◉ Não **27 - Quantos dias durou a dor de garganta?**

28 - Nas últimas duas semanas, isto é, desde (DIA DA SEMANA) da semana retrasada, (NOME) TOSSE:

◉ Sim ◉ Não

◉ Tem sempre/constantemente **29 - A tosse era com catarro?**

◉ Sim ◉ Não **30 - Começou há quantos dias?**

31 - Ainda tem tosse?

◉ Sim ◉ Não

32 - Quantos dias durou a tosse?

33- Nas últimas duas semanas, isto é, desde (DIA DA SEMANA) da semana retrasada, (NOME) DIFICULDADE DE RESPIRAR:

◉ Sim ◉ Não

◉ Tem sempre/constantemente **33**

- O nariz estava entupido?

◉ Sim ◉ Não **34 -**

Começou há quantos dias?

____

35 - Ainda tem dificuldade de respirar?

◉ Sim ◉ Não **36 - Quantos dias durou a**

dificuldade de respirar?

____

37 - Nas últimas duas semanas, isto é, desde (DIA DA SEMANA) da semana retrasada, (NOME) teve PALPITAÇÃO, CORAÇÃO DISPARADO OU BATEDEIRA NO CORAÇÃO:

◉ Sim ◉ Não

◉ Tem sempre/constantemente

38 - Começou há quantos dias?

____

39 - Ainda tem esse problema no coração?

◉ Sim ◉ Não **40 - Quantos dias durou a**

palpitação no coração?

____

41 - Nas últimas duas semanas, isto é, desde (DIA DA SEMANA) da semana retrasada, (NOME) PASSOU A SENTIR MENOS O CHEIRO OU O GOSTO DAS COISAS?

Sim Não

42 - Começou há quantos dias?

____

43 - Ainda está tendo esse problema?

Sim Não **44 - Quantos dias ficou sem o cheiro ou o gosto das coisas?**

____

Sim

Não

45 - Nas últimas duas semanas, isto é, desde (DIA DA SEMANA) da semana retrasada, (NOME) teve DIARREIA:

46 - Começou há quantos dias?

____

47 - Ainda está com diarreia?

Sim Não **48 - Quantos dias durou a diarreia?**

____

49 - Nas últimas duas semanas, isto é, desde (DIA DA SEMANA) da semana retrasada, (NOME) teve VÔMITOS:

Sim Não **50 - Começou há quantos dias?**

____

51 - Ainda tem vômitos?

Sim Não **52 - Quantos dias duraram os vômitos?**

53 - Algum médico já disse que (NOME) tem

	Sim	Não
Hipertensão ou pressão alta?	◉	◉
Diabetes ou açúcar no sangue?	◉	◉
Asma ou bronquite?	◉	◉
Câncer (qualquer tipo)?	◉	◉
Doença crônica nos rins?	◉	◉
Alguma doença no coração?	◉	◉
Outra doença crônica?	◉	◉

54 - (NOME) foi a algum serviço de saúde nos últimos 15 dias? Sim Não

◉ ◉

55 - Que tipo de serviço de saúde?

◉ Postinho

◉ Pronto Atendimento ou Pronto Socorro ◉ Médico particular

◉ Hospital

◉ Outro serviço

56 - Por qual motivo procurou o serviço?

◉ Vacinação

◉ Problema de saúde já existente

◉ Retorno agendado

◉ Sintomas de gripe ou dor de garganta

◉ Buscar remédio

◉ Outro motivo

57 - Com relação ao distanciamento social que está sendo orientado pelas autoridades de saúde, ou seja, ficar em casa e evitar contato com outras pessoas, quanto (NOME) acha que está conseguindo fazer? (ler as alternativas)

◉ Muito pouco ◉ Pouco

◉ Mais ou menos ◉ Bastante

◉ Praticamente isolado de todo mundo

58 - Como tem sido a rotina de atividades de (NOME)? (ler as alternativas)

◉ Fica em casa o tempo todo

◉ Sai apenas para coisas essenciais como comprar comida ◉ Sai de vez em quando para compras e esticar as pernas ◉ Sai todos os dias para alguma atividade

◉ Sai todos os dias, o dia todo, para trabalhar ou outra atividade regular

59 - Pensando na rotina da casa, quem tem entrado na casa? (ler as alternativas)

◉ Só os familiares que moram junto, se tiver, e mais ninguém

◉ Alguns parentes próximos visitam 1 a 2 vezes por semana

◉ Alguns parentes próximos visitam quase que todos os dias

◉ Amigos e parentes visitam 1 a 2 vezes por semana

◉ Amigos e parentes visitam quase todos os dias

60 - IDENTIFICAÇÃO DO TESTE: (Nº: RODADA+MUNICÍPIO+SETOR+DOMICILIO) (LER EM VOZ ALTA)

---

65 - RESULTADO DO TESTE:

- ◉ POSITIVO
- ◉ NEGATIVO
- ◉ INCONCLUSIVO

66 - SE POSITIVO, MARQUE OS MORADORES QUE SERÃO TESTADOS: (VER QUADRO DO DOMICÍLIO)

- ☑ Morador 1
- ☑ Morador 2
- ☑ Morador 3
- ☑ Morador 4
- ☑ Morador 5
- ☑ Morador 6
- ☑ Morador 7
- ☑ Morador 8
- ☑ Morador 9
- ☑ Morador 10
- ☑ Morador 11
- ☑ Morador 12
- ☑ Morador 13
- ☑ Morador 14
- ☑ Morador 15

68 - Nome do entrevistado(a):

69 - Telefone para contato:

( )______-_____
