# Supplementary material for: Insufficient social distancing may contribute to COVID-19 outbreak: The case of Ijuí city in Brazil
Source: PLoS One. 2021 Feb 17;16(2):e0246520. doi: 10.1371/journal.pone.0246520 (PMC7888680; doi:10.1371/journal.pone.0246520)
Supplement: Attachment — Submitted filename: Comments and suggestions about paper Covid-19 14 10 2020.docx [file pone.0246520.s002.docx]

Ref.: Insufficient social distancing may be related to COVID-19 outbreak: the case of Ijuí city (RS) in Brazil

Comments and suggestions:

The study seems to me to be well written, with the data well summarized and presenting and discussing a set of data can be interesting to understand the role of social distance in the management, mitigation, of the pandemic outbreak, particularly in the case of the city of Ijuí, a of the most populous in the state of Rio Grande do Sul, Brazil.

However, I would like to point out some specific and general aspects and comments, which I understand, could enrich and clarify the content of the study.

Specific comments:

1. In the introductory part of the manuscript, starting at line 54, it would be interesting for the authors to update the data by comparing some indicators of cases and deaths, especially with other Brazilian states. Perhaps, with the States of São Paulo, Rio de Janeiro and Ceará for which various indicators have always been the highest in Brazil. Perhaps, an illustrative table or figure can be used showing a relative comparative configuration of the varied incidence indicators (free choice by the authors);

2. It would also be interesting to describe and characterize Brazil, as a whole <showing that it is a nation with 27 states (including the DF) and that the timing and actions against the pandemic were very uneven. Perhaps, it is also important to report, albeit very quickly, the uncertainty that affected Brazil, including government ambiguity and health agents throughout the pandemic. The effects of this uncertainty and confusion, as well as the sense of urgency, are important dimensions that affect the social distance supposedly undertaken by agents of pandemic control, especially from the government;

3. It is also interesting to show how the indicators of social distance, control and measurement, varied weekly (choose the unit of analysis), or over weekends when compared to working days. I recommend, see Prof. Steven Taylor, Psychology of Pandemics, .... 2020);

4. From line 71, I believe that the description of the distance is insufficient. I recommend doing a brief review of the literature (there are several papers on this topic) about the role of social detachment, social isolation and other behavioral measures (the only ones that are effective while the vaccine or other collective immunization process is absent). The literature and the importance highlighted between lines 71 to 78 are quite limited in view of the large number of studies (papers) hitherto published on the role of isolation and social distance in controlling the spread of Covid-19;

5. Between lines 112 to 121, one could (if not inserted before) a table comparing the weekly variation or Regina comparison within the State of RS of the social distancing indicator (MSDI), as well as on its validity and reliability ( accuracy, reliability, as a behavioral indicator);

6. Somewhere in the description and characterization of the sample, population studied, it could be, in my best consideration, insert a table describing the population characteristics of those considered in the studies in some of its phases, especially responding to surveys, questionnaires and other sociodemographic information. Brazil and its 27 states are very unequal considering any indicator of inequality, for example, the GINI index or even HDI-regional or state, municipal;

General comments:

1. I think that the authors should consider some other variables that may be underlying the effects of social detachment, or social isolation, such as the personality variables of individuals and the social psychological variables that affect, overly, the attitudes and behaviors related to hygienic practices, cognitive assessment of risk factors, social distance and social isolation, as well as when to implement, if this occurs, mass, collective vaccination.

2. Spatial distance, temporal distance, social distance and probability distance (heuristic availability) could be considered in passing in the analysis of the results, especially in the discussion. There is an immense literature showing that these psychological distances affect the perceived risk and affect the cognitive assessment of the importance of the immediate introduction of distance and social isolation;

3. Data analysis is clearly described and summarized in figures that are very revealing;

4. Could the authors discuss the downward trend in the social distancing indicators that are becoming evident as the pandemic outbreak has lengthened? Was there a psychological accommodation, or a pandemic fatigue?

5. I understand that the role of social detachment should at least be discussed in the important light of psychological factors of personality and psychological social factors. Behaviors are the genuine tools we have to manage and mitigate the pandemic outbreak;

6. Statistical and epidemiological analyzes are appropriate and well described;

7. The work, in my view, constitutes an important study to understand the pandemic spread.

And my opinion,

October 14, 2020

José Aparecido Da Silva

Full Professor
