# Supplementary material for: Insufficient social distancing may contribute to COVID-19 outbreak: The case of Ijuí city in Brazil
Source: PLoS One. 2021 Feb 17;16(2):e0246520. doi: 10.1371/journal.pone.0246520 (PMC7888680; doi:10.1371/journal.pone.0246520)
Supplement: Attachment — Submitted filename: Insufficient social distancing may contribute to COVID final review.doc [file pone.0246520.s004.doc]

Insufficient social distancing may contribute to COVID-19 outbreak: the case of Ijuí city in Brazil

Short Title: Social distancing and COVID-19 outbreak in Ijuí-Brazil

Corresponding Author: Thiago Gomes Heck, Ph.D. University of Nortwestern Rio Grande do Sul State (UNIJUI) Ijuí, RS BRAZIL

-----------------------

Final Opinion:

Dear Editor:

I read the manuscript reviewed by the authors very carefully. I could see the care, the attention, the care with which the authors reviewed the manuscript. They responded, essentially and in depth, to all the suggestions and comments we made during our first review. Importantly, the authors were very humble and didactic in their responses. I was really very pleased to participate in this review process in which, for me, there was scientific respect for peers and the ethics that must prevail in the interaction between researchers.

The manuscript is very enriched, presents a lot of details and faithfully portrays the scenario of distance (social, physical) in a large city in Brazil

The authors should be congratulated for the excellent work and review.

Te best wishes
